# Gender Differences in Epicardial Adipose Tissue and Plaque Composition by Coronary CT Angiography: Association with Cardiovascular Outcome

**DOI:** 10.3390/diagnostics13040624

**Published:** 2023-02-08

**Authors:** Ullrich Ebersberger, Maximilian J. Bauer, Florian Straube, Nicola Fink, U. Joseph Schoepf, Akos Varga-Szemes, Tilman Emrich, Joseph Griffith, Ellen Hoffmann, Christian Tesche

**Affiliations:** 1 Department of Cardiology, Munich University Clinic, Ludwig-Maximilians-University, 80636 Munich, Germany; 2 Department of Cardiology and Intensive Care Medicine, Heart Center Munich-Bogenhausen, 81925 Munich, Germany; 3 Kardiologie München Nord, 80939 Munich, Germany; 4 Division of Cardiovascular Imaging, Department of Radiology and Radiological Science, Medical University of South Carolina, Charleston, SC 29425, USA; 5 Department of Radiology, University Hospital, Ludwig-Maximilians-University, 81377 Munich, Germany; 6 Division of Cardiology, Medical University of South Carolina, Charleston, SC 29425, USA; 7 Department of Diagnostic and Interventional Radiology, University Medical Center Mainz, 55131 Mainz, Germany; 8 German Center for Cardiovascular Research (DZHK), Partner Site Rhine-Main, 55131 Mainz, Germany; 9 Department of Cardiology, Augustinum Clinic Munich, 81375 Munich, Germany

**Keywords:** coronary computed tomography angiography, coronary artery disease, cardiovascular outcome, gender, plaque quantification, epicardial adipose tissue

## Abstract

**Background:** To investigate gender differences in epicardial adipose tissue (EAT) and plaque composition by coronary CT angiography (CCTA) and the association with cardiovascular outcome. **Methods:** Data of 352 patients (64.2 ± 10.3 years, 38% female) with suspected coronary artery disease (CAD) who underwent CCTA were retrospectively analyzed. EAT volume and plaque composition from CCTA were compared between men and women. Major adverse cardiovascular events (MACE) were recorded from follow-up. **Results:** Men were more likely to have obstructive CAD, higher Agatston scores, and a larger total and non-calcified plaque burden. In addition, men displayed more adverse plaque characteristics and EAT volume compared to women (all *p* < 0.05). After a median follow-up of 5.1 years, MACE occurred in 8 women (6%) and 22 men (10%). In multivariable analysis, Agatston calcium score (HR 1.0008, *p* = 0.014), EAT volume (HR 1.067, *p* = 0.049), and low-attenuation plaque (HR 3.82, *p* = 0.036) were independent predictors for MACE in men, whereas only low-attenuation plaque (HR 2.42, *p* = 0.041) showed predictive value for events in women. **Conclusion:** Women demonstrated less overall plaque burden, fewer adverse plaque characteristics, and a smaller EAT volume compared to men. However, low-attenuation plaque is a predictor for MACE in both genders. Thus, a differentiated plaque analysis is warranted to understand gender differences of atherosclerosis to guide medical therapy and prevention strategies.

## 1. Introduction

Coronary CT angiography (CCTA) has evolved to the standard of care for the non-invasive evaluation of coronary artery disease (CAD) and plaque quantification [1,2,3]. Previous studies have shown the predictive ability of coronary plaque measurements (i.e., plaque burden, composition, and high-risk features) for improved risk stratification [4,5,6]. 

In addition, information derived from detailed plaque analysis enables tailored and individualized medical therapy and prevention [7]. Epicardial adipose tissue (EAT) derived from CCTA imaging has been proposed as a novel marker of coronary vascular inflammation and promotor of atherosclerosis with prognostic value for major adverse cardiovascular events (MACE) [8]. Thus, EAT volume is considered a non-invasive tool which can refine risk stratification of patients with CAD for improved prognostication [9,10,11].

Gender differences in CAD are well established and a major focus of ongoing research. Women show different risk factor profiles, symptoms, plaque composition, and prognosis compared to men [12]. These differences culminate in fewer interventional procedures and less evidence-based medical treatment and preventive care in women in comparison to men [13,14]. Therefore, detailed and tailored quantitative plaque analysis is required for gender-specific risk stratification and appropriate treatment strategies to overcome the limitations of a solely risk score based estimation. 

Thus, we sought to investigate gender differences in EAT volume and plaque composition by CCTA and the association with cardiovascular outcome.

## 2. Material and Methods

### 2.1. Study Population

The protocol of this retrospective, single-center study and a waiver of informed consent were approved by the Institutional Review Board in compliance with the Health Insurance Portability and Accountability Act (HIPAA) of 1996. Data of consecutive patients with suspected CAD who had undergone CCTA between April 2009 and August 2013 were retrospectively analyzed. General patient characteristics, cardiovascular risk factors and comorbidities were obtained from electronic medical reports. MACE were recorded on follow-up and defined as: (i) cardiac death, (ii) nonfatal myocardial infarction and (iii) unstable angina leading to coronary revascularization at least 6 weeks after CCTA [15]. Patients were excluded if they were diagnosed with acute coronary syndrome during the time of care involving the CCTA scan, underwent coronary revascularization within six weeks of the CCTA scan, or had a history of previous coronary intervention or coronary artery bypass graft procedure. In addition, patients with missing or non-diagnostic CCTA image quality were excluded from further evaluation.

### 2.2. CCTA Acquisition

Imaging was performed using a sixty-four-slice CT (Philips Brilliance 64, Philips Medical, Eindhoven, The Netherlands). First, patients underwent a non-contrast enhanced calcium scoring scan (collimation, 32 × 1.2 mm^2^; 120 kV tube voltage; tube current, 75 mA; 3 mm slice thickness with 1.5 mm increment). Thereafter, a retrospectively ECG-gated acquisition in spiral technique was used for the subsequent contrast-enhanced CCTA (120 kV tube voltage, 600 mAs tube current, temporal resolution of 165 ms, collimation 64 × 0.6 mm^2^). Contrast enhancement was performed by injecting 50–80 mL of iodine contrast agent at 4–6 mL/s followed by a 30 mL saline bolus chaser. The attending physician of the day individually determined the use of beta-blockers and nitroglycerin. Weighted filtered back projection image reconstruction was performed in the cardiac phase with the least motion with a section thickness of 0.75 mm, reconstruction increment of 0.5 mm, and a smooth convolution kernel.

### 2.3. Analysis of CCTA Data, Plaque Parameters, and EAT Volume

cCTA data were evaluated on a post-processing workstation (Philips Medical, Eindhoven, The Netherlands). Two observers experienced in cCTA analysis who were blinded to the patients’ histories analyzed the lesion characteristics independently. All discordant cases were resolved with a consensus reading. For stenosis grading the CAD-RADS™ (coronary artery disease reporting and data system) was used: 1. none (0%) or minimal (1–24%), 2. mild (25–49% stenosis), 3. moderate (50–69% stenosis), 4. severe (70–99% stenosis), 5. total occlusion (100%). Obstructive CAD was defined as ≥50% luminal stenosis [16]. Plaque composition and morphology was assessed using a dedicated, semi-automated plaque software (Syngo.Via Frontier, version 4.2.0, Siemens Healthineers, Forchheim, Germany) with prespecified Hounsfield unit (HU)-based cut-off values for each plaque component: calcified (>350 HU), fibrous (30–350 HU), and lipid (<30 HU) [17]. EAT volume was measured on non-contrast enhanced images as previously described in detail [18,19]. In brief, EAT was defined as fat contained within the pericardium and quantified using a region of interest (ROI) manually defined by tracing the pericardium from the pulmonary artery trunk to the apex of the heart in axial sections at 1 cm intervals. EAT was calculated as the sum of all pixels within a window of −190 to −30 HU in the ROI.

Plaques with a CT attenuation of ≤30 Hounsfield units (HU) were defined as low-attenuation plaques. On vessel cross-sections, the presence of positive vessel remodeling was measured as the ratio of the vessel area of the lesion over the proximal luminal reference area. A remodeling index ≥1.1 was defined as positive vessel remodeling. The presence of a positive napkin-ring sign, described as a low attenuating plaque core circumscribed by an area of higher attenuation, was assessed. High-risk plaque features, including low-attenuation plaque, napkin-ring sign, positive remodeling, and spotty calcifications, were assessed [20,21].

### 2.4. Statistical Analysis

MedCalc (MedCalc Software, version 15, Ostend, Belgium) and SPSS Statistics (IBM, SPSS, Version 23) were used for all statistical analyses. Continuous variables are shown as mean ± standard deviation or median with interquartile range when not normally distributed. The Student *t*-test and Mann–Whitney *U*-test were used for parametric and non-parametric data, respectively. EAT volume was adjusted according to the body surface area as indexed EAT volume (cm^3^/m^2^). The predictive value of parameters for MACE was assessed for men and women using univariable and multivariable Cox proportional hazards analysis with backward elimination based on *p*-values as selection criterion. Hazard ratios (HR) and 95% confidence intervals are displayed. To avoid overfitting, a multivariable model was built utilizing recursive feature elimination with 5-fold cross-validation and C-indices as performance criterion. MACE were calculated by Kaplan–Meier curves and compared with log-rank test in men and women according to EAT volume (using the median of ≥50.8 vs. <50.1 for men and the median of ≥47.7 vs. <47.7 for women), and presence of low-attenuation plaque (yes/no). Statistical significance was assumed with a *p*-value ≤0.05. 

## 3. Results

### 3.1. Patient Characteristics

The study population consisted of 352 patients (64.2 ± 10.3 years, 38% female) who had undergone CCTA for the assessment of CAD. Women were older and had higher body mass index compared to men (both *p* < 0.05). However, there was no statistically significant difference in terms of cardiovascular risk factors. The Framingham risk score showed no significantly different scores for cardiovascular risk in men (10.4 [IQR 8.2, 13.6] in comparison to women (10.2 [IQR 7.9, 13.1, *p* = 0.65]. The baseline characteristics of the study population are shown in Table 1.

### 3.2. Quantification of EAT Volume and CCTA Plaque Parameters 

Women had lower Agatston calcium scores compared to men (median 2.8 vs. median 17.5, *p* = 0.005) and were less likely to have obstructive CAD (25% vs. 37%, *p* = 0.019). 

EAT volume (adjusted to the body surface) was significantly higher in men than in women (median 50.8 [IQR 44.6, 56.8] vs. median 47.7 [IQR 43.2, 53.2], *p* = 0.004). Visually assessed adverse plaque characteristics (low-attenuation plaque, positive remodeling, napkin-ring sign, and ≥2 high-risk features) were significantly more frequent in men than in women (all *p* < 0.05). Only spotty calcifications showed no relevant difference between genders (24% vs. 18%, *p* = 0.21). 

Quantitative plaque analysis revealed significantly different plaque volumes in men for total plaque volume (median 119.4 [IQR 64.2, 182.3]) and non-calcified plaque volume (median 90.2 [IQR 45.4, 138.2]) compared to women (total plaque volume: median 100.2 [IQR 76.7, 137.3], non-calcified plaque volume: median 79.7 [IQR 43.0, 117.1], all *p* < 0.05). However, calcified plaque volume showed no significant difference between genders (*p* = 0.76) (Table 2). 

### 3.3. Association of EAT Volume and Plaque Parameters with MACE

In the median follow-up of 5.1 years (IQR 4.9–5.4 years) MACE occurred in 8 women (6%) and 22 men (10%). Both women and men who experienced MACE demonstrated higher adverse plaque characteristics (low-attenuation plaque, positive remodeling, napkin-ring sign, and ≥2 high-risk features) compared to those of the same gender without events. However, total plaque volume and presence of obstructive CAD were only significantly different in men, but not in women, who suffered MACE when compared to the event-free cohort of the same gender, respectively. In addition, EAT volume was significantly higher in women (53.0 [IQR 45.3, 59.8]) and men (57.8 [IQR 53.2, 64.4]) with MACE when compared to patients with no adverse events (women: 47.6 [IQR 42.5, 53.1]), men: 50.3 [IQR 44.6, 55.8], all *p* < 0.05) (Table 3). 

**Figure 1:**
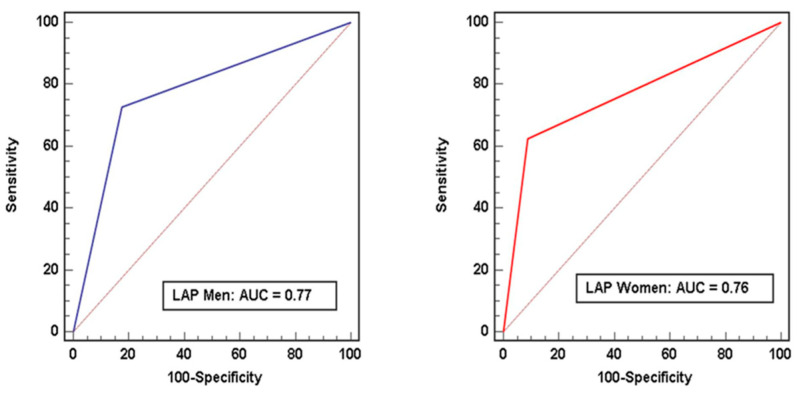
Diagnostic performance of low-attenuation plaque for the prediction of MACE in men and women. Receiver operating characteristics curves with concordant C-indices revealed a C-index of low-attenuation plaque of 0.77 (95%CI 0.72–0.83, *p* < 0.001) in men and 0.76 (95%CI 0.68–0.84, *p* = 0.004) in women for outcome prediction. LAP = low-attenuation plaque.

The following parameters were independent predictors for MACE in multivariable analysis in men: Agatston calcium score (HR 1.0008, [95% CI 1.0002–1.0014], *p* = 0.014), EAT volume (HR 1.067, [95% CI 1.001–1.14], *p* = 0.049), and the presence of low-attenuation plaque (HR 3.82, [95% CI 1.02–14.34], *p* = 0.036). In women, only the presence of low-attenuation plaque (HR 2.42, [95% CI 1.06–11.75], *p* = 0.041) remained an independent predictor for MACE (Table 4). Low-attenuation plaque revealed a C-index of 0.77 (95%CI 0.72–0.83, *p* < 0.001) in men and 0.76 (95%CI 0.68–0.84, *p* = 0.004) in women for outcome prediction using ROC analysis (Figure 1). 

**Figure 2:**
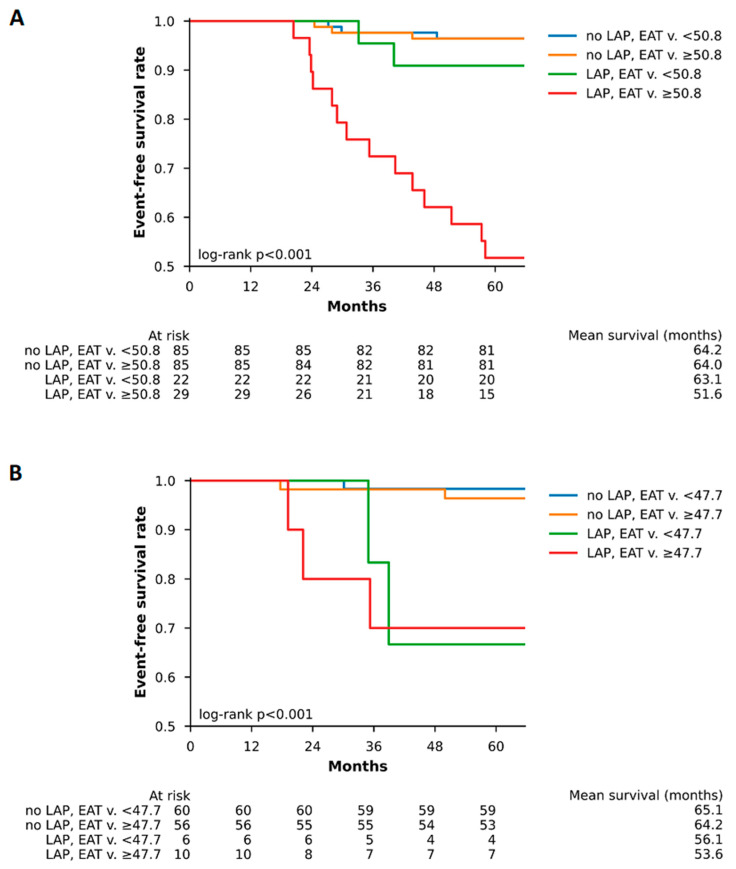
Kaplan–Meier curves in men and women according to the presence of low-attenuation plaque and EAT volume. Kaplan–Meier curves stratified by a combination of the presence of low-attenuation plaque and EAT volume in (**A**) men and (**B**) women. EAT = epicardial adipose tissue, LAP = low-attenuation plaque.

The Kaplan–Meier survival curves for men and women according to presence of low-attenuation plaque and EAT volume are displayed in Figure 2. Men and women with low-attenuation plaques and above median EAT volume experienced significantly more events than the group without these markers (*p* < 0.001). 

## 4. Discussion

In the present study we evaluated gender differences in EAT volume and plaque composition by CCTA and the subsequent association with cardiovascular outcome. Our findings support the importance of EAT volume, plaque composition, and the presence of adverse plaque characteristics for the ability to improve risk prediction of MACE in both men and women. Agatston calcium score (HR 1.0008, *p* = 0.014), EAT volume (HR 1.067, *p* = 0.049), and low-attenuation plaque (HR 3.82, *p* = 0.036) were independent predictors for MACE in men, whereas low-attenuation plaque (HR 2.42, *p* = 0.041) showed predictive value for cardiovascular events in women. These markers may help improve risk-stratification by gender to guide medical therapy and prevention strategies. 

Several prior studies have examined gender differences in coronary artery plaque formation to provide better gender-specific therapy [13,14,22,23]. A recent analysis of the SCOT-HEART (Scottish Computed Tomography of the Heart) study showed that women have less coronary plaque burden and subsequent MACE compared to men [14]. In addition, the authors found that the existence of low-attenuation plaque was an important predictor for events. Likewise, Plank et al. [13] and Senoner et al. [23] demonstrated that plaque burden and presence of high-risk plaque features were higher in men compared to women and that high-risk plaques had predictive value for MACE. 

In line with these findings, we also found significant differences for severity of CAD and plaque burden in men and women (obstructive CAD 37% vs. 25%, *p* = 0.019, median total plaque volume 119.4 vs. 100.2, *p* = 0.016). More importantly, in both men and women who suffered MACE, high-risk plaque features and EAT volume, which function as a substitute of pericoronary inflammation and promotor of coronary plaque, were significantly higher when compared to the group without events. The presence of low-attenuation plaque was the strongest predictor for adverse events in both genders. In addition, the Kaplan–Meier analysis provides a quality visualization of the significantly higher incidence of MACE in both men and women who have low-attenuation plaques and greater than median EAT volumes. A sub-study of the PROMISE (Prospective Multicenter Imaging Study for Evaluation of Chest Pain) trial [5] and the ICONIC study (Incident COronary EveNts Identified by Computed Tomography) [24] used quantitative plaque analysis to show a strong association between plaque composition and high-risk plaque features with adverse cardiovascular events in men and women. However, they did not assess the impact of EAT which is a novel biomarker for MACE prediction. Our results are in accordance with the previously mentioned findings and go beyond their results as we showed that high-risk low-attenuation plaque and EAT volume were significantly different in both genders and especially in patients with MACE. The interplay and pathophysiological mechanism between EAT, pericoronary inflammation, and the process of atherosclerosis and adverse plaque characteristics are not yet fully understood [25]. In addition, endogenous hormonal factors, such as estrogen, have an impact on lipid metabolism and endothelial function and might promote atherosclerotic plaque progression in women. Women are still underdiagnosed and undertreated in terms of CAD which may account for higher mortality rates [26]. This finding is supported by a report from the European Heart Survey on stable angina pectoris which indicates that women undergo less non-invasive ischemia testing or invasive coronary angiography and subsequent revascularization procedures [27]. 

Despite plaque morphology and composition, the clinical presentation and symptom typicality has an impact on cardiovascular outcome in both men and women. Recent data of the CONFIRM (COroNary CT Angiography Evaluation for Clinical Outcomes) registry have demonstrated increased risk of adverse events associated with typical chest pain when compared to asymptomatic patients or patients with atypical or non-cardiac chest pain [28]. We also found significant differences in terms of clinical presentation at admission in our study population. Men presented more often with typical chest pain, whereas women had more atypical chest pain.

Gender differences in the atherosclerotic profile of men and women can be adequately visualized by quantitative plaque analysis using CCTA. The challenge and the focus for further investigations is to transfer this knowledge into clinical practice for tailored pharmacological and preventive care with respect to gender. 

Some limitations of this study need to be addressed. This is a retrospective investigation which is therefore subject to limitations inherent to this study design. A relatively small number of patients were included, which may incur selection bias. Prospective studies in larger study cohorts will be necessary to validate our findings. Therefore, the data generated in this study should only be hypothesis generating. We did not include the typicality of clinical presentation in the outcome analysis due to the small sample size. However, it is well-known that clinical symptoms of chest pain have prognostic value [28,29]. Our results on multivariable analysis may be underpowered by the limited number of observations per variable included [30]. Patient follow-up was performed using electronic medical records of the hospitals; therefore, we might have missed events occurring outside the hospital. 

In conclusion, our study demonstrates that women have less overall plaque burden, fewer adverse plaque characteristics, and smaller EAT volumes with lower risk of adverse cardiovascular events in comparison to men. Nevertheless, high-risk low-attenuation plaque is a strong predictor for MACE in both genders. Thus, a differentiated plaque analysis is warranted to understand gender differences of atherosclerosis in order to guide medical therapy and prevention strategies. 

## Figures and Tables

**Table 1:** Patient demographics. Total patient cohort (*n* = 352).

Parameter	Male (*n* = 220)	Female (*n* = 132)	*p* Value
Age (years)	60.3 ± 10.1	66.4 ± 10.5	0.001
Body-mass-index (kg/m^2^)	27.8 ± 5.7	28.8 ± 4.9	0.002
Hypertension *n* (%)	120 (54%)	74 (56%)	0.74
Dyslipidemia *n* (%)	95 (43%)	60 (45%)	0.65
Tobacco abuse *n* (%)	65 (29%)	31 (24%)	0.23
Diabetes mellitus *n* (%)	41 (18%)	21 (15%)	0.89
CAD family history *n* (%)	61 (28%)	34 (26%)	0.71
Framingham risk score	10.4 (8.2, 13.6)	10.2 (7.9, 13.1)	0.65
Medication at admission			
Aspirin	74 (33%)	38 (29%)	0.48
Statins	90 (41%)	50 (38%)	0.72
Betablocker	48 (22%)	24 (18%)	0.54
ACE inhibitor	41 (19%)	22 (17%)	0.49
Diuretics	44 (20%)	21 (16%)	0.67
Clinical presentation at admission			
No chest pain	106 (48%)	54 (41%)	0.04
Non-cardiac chest pain	31 (14%)	24 (18%)	
Typical chest pain	38 (17%)	14 (11%)	
Atypical chest pain	64 (29%)	41 (31%)	

Data presented as mean ± standard deviation, as medians with 25th and 75th percentile or percentages in parentheses (%). CAD = coronary artery disease. ACE = angiotensin converting enzyme.

**Table 2:** Findings of quantitative analysis of coronary CT markers in men and women.

Parameter	Male (*n* = 220)	Female (*n* = 132)	*p* Value
Agatston score	17.5 (0.0, 142.1)	2.8 (0.0, 58.9)	0.005
Obstructive CAD	82 (37%)	33 (25%)	0.019
EAT volume (cm^3^/m^2^)	50.8 (44.6, 56.8)	47.7 (43.2, 53.2)	0.004
Total plaque volume (mm^3^)	119.4 (64.2, 182.3)	100.2 (67.7, 137.3)	0.016
Calcified plaque volume (mm^3^)	26.7 (4.3, 67.0)	26.1 (5.3, 65.3)	0.76
Non-calcified plaque volume (mm^3^)	90.2 (45.4, 138.2)	79.7 (43.0, 117.1)	0.02
Low-attenuation plaque	51 (23%)	16 (12%)	0.011
Spotty calcification	55 (24%)	24 (18%)	0.21
Positive remodeling	65 (29%)	26 (19%)	0.043
Napkin-ring sign	36 (16%)	11 (8%)	0.033
≥2 high-risk plaque features	54 (24%)	17 (13%)	0.007
1 vessel CAD	39 (18%)	11 (8%)	0.01
2 vessel CAD	24 (11%)	5 (4%)	
3 vessel CAD	11 (5%)	2 (2%)	

Data presented as medians with 25th and 75th percentile or numbers with percentages (%). CAD = coronary artery disease. EAT = epicardial adipose tissue.

**Table 3:** Findings of quantitative analysis of coronary CT markers in men and women according to MACE.

Male			
Parameter	MACE (*n* = 22)	No MACE (*n* = 198)	*p* Value
Agatston score	690 (181, 2112)	11 (1.0, 97.3)	<0.001
Obstructive CAD	19 (86%)	63 (32%)	<0.001
EAT volume (cm^3^/m^2^)	57.8 (53.2, 64.4)	50.3 (44.6, 55.8)	0.002
Total plaque volume (mm^3^)	129.4 (94.2, 153.5)	112.0 (58.8, 184.9)	0.034
Calcified plaque volume (mm^3^)	11.4 (3.5, 54.1)	29.4 (3.2, 67.0)	0.57
Non-calcified plaque volume (mm^3^)	115.8 (94.9, 156.3)	89.6 (44.8, 136.7)	0.037
Low-attenuation plaque	16 (72%)	35 (17%)	<0.001
Spotty calcification	13 (59%)	40 (20%)	<0.001
Positive remodeling	13 (59%)	52 (26%)	0.015
Napkin-ring sign	11 (50%)	25 (13%)	<0.001
≥2 high-risk plaque features	15 (68%)	40 (20%)	<0.001
**Female**			
**Parameter**	**MACE (*n* = 8)**	**No MACE (*n* = 124)**	***p *** **Value**
Agatston score	12.1 (59.9, 389.5)	1.2 (0.0, 48.0)	0.003
Obstructive CAD	6 (75%)	27 (22%)	0.82
EAT volume (cm^3^/m^2^)	53.0 (45.3, 59.8)	47.6 (42.5, 53.1)	0.018
Total plaque volume (mm^3^)	147.3 (112.1, 171.6)	98.8 (63.9, 132.8)	0.018
Calcified plaque volume (mm^3^)	10.7 (5.3, 51.6)	30.7 (5.3, 65.3)	0.74
Non-calcified plaque volume (mm^3^)	119.9 (82.9, 141.3)	77.3 (42.6, 115.9)	0.041
Low-attenuation plaque	5 (63%)	11 (9%)	<0.001
Spotty calcification	5 (63%)	19 (15%)	0.008
Positive remodeling	5 (63%)	21 (17%)	0.002
Napkin-ring sign	3 (38%)	8 (6%)	0.002
≥2 high-risk plaque features	6 (75%)	11 (9%)	<0.001

Data presented as medians with 25th and 75th percentile or numbers with percentages (%); EAT = epicardial adipose tissue. CAD = coronary artery disease.

**Table 4:** Univariable and multivariable Cox proportional hazards regression analysis of coronary CT-derived parameters in men and women for the prediction of MACE.

Parameter	Univariable Analysis Hazard Ratio 95% CI *p* Value			Multivariable Analysis Hazard Ratio 95% CI *p* Value		
Male						
Agatston score	1.001	1.007–1.002	0.001	1.0008	1.0002–1.0014	0.014
Obstructive CAD	13.67	3.91–47.93	0.001	-	-	-
EAT volume	1.09	1.04–1.15	0.007	1.067	1.001–1.14	0.049
Total plaque volume	1.00	0.99–1.006	0.69	-	-	-
Calcified plaque volume	0.99	0.98–1.005	0.53	-	-	-
Non-calcified plaque volume	1.004	0.99–1.01	0.15	-	-	-
Low-attenuation plaque	12.49	4.56–34.19	0.001	3.82	1.02–14.34	0.036
Spotty calcification	5.74	2.29–14.37	0.002	-	-	-
Positive remodeling	4.08	1.65–10.11	0.002	-	-	-
Napkin-ring sign	6.96	2.73–17.73	0.001	-	-	-
≥2 high-risk features	8.52	3.26–22.28	0.001	-	-	-
**Female**						
Agatston score	1.001	0.99–1.002	0.26	-	-	-
Obstructive CAD	10.7	2.05–56.4	0.004	-	-	-
EAT volume	1.08	0.99–1.18	0.06	-	-	-
Total plaque volume	1.01	1.001–1.02	0.04	-	-	-
Calcified plaque volume	1.007	0.99–1.01	0.12	-	-	-
Non-calcified plaque volume	1.02	0.99–1.03	0.08	-	-	-
Low-attenuation plaque	17.12	3.59–81.4	0.004	2.42	1.06–11.75	0.041
Spotty calcification	9.21	2.03–41.78	0.004	-	-	-
Positive remodeling	8.17	1.81–36.86	0.006	-	-	-
Napkin-ring sign	8.70	1.75–43.12	0.008	-	-	-
≥2 high-risk features	30.81	5.54–171.42	0.001	-	-	-

## Data Availability

The data underlying this article will be shared on reasonable request to the corresponding author.

## Abbreviations

AUC	Area under the curve
CAD	Coronary artery disease
CAD-RADS™	Coronary artery disease reporting and data system
CCTA	Coronary CT angiography
EAT	Epicardial adipose tissue
HR	Hazard ratio
HU	Hounsfield unit
IQR	Interquartile range
MACE	Major adverse cardiovascular events
PCAT	Pericoronary adipose tissue
RI	Remodeling Index
ROC	Receiver-operating characteristics
ROI	Region of interest
